# Sarcoidosis-lymphoma syndrome: a diagnostic dilemma

**DOI:** 10.36416/1806-3756/e20240105

**Published:** 2024-06-21

**Authors:** Clara Perini Fiorot, Rosana Souza Rodrigues, Edson Marchiori

**Affiliations:** 1. Universidade Federal do Rio de Janeiro, Rio de Janeiro (RJ) Brasil.; 2. Instituto D’Or de Pesquisa e Ensino - IDOR - Rio de Janeiro (RJ) Brasil.

**Figure 1:**
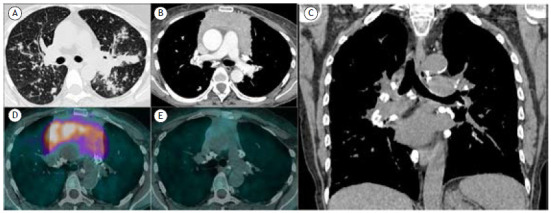
In A, an axial CT image of the chest (lung window) showing small nodules with peribronchovascular infiltrations. Mediastinal lymph node enlargement was also observed (not shown). In B, axial CT image (mediastinal window) showing a large mass with soft-tissue density in the anterior mediastinum. In C, a coronal reconstruction image (mediastinal window) showing the mediastinal mass and lymph node calcifications in multiple hilar and mediastinal chains. In D, axial PET/CT fusion image showing diffusely high ^18^F-fluorodeoxyglucose (^18^F-FDG) uptake of the mass. In E, PET/CT image acquired 10 months after treatment initiation showing marked reduction of the mass volume and absence of ^18^F- FDG uptake. Note also mediastinal lymph node calcifications.

A 57-year-old patient who had been diagnosed with pulmonary sarcoidosis 10 years prior and had received irregular corticosteroid treatment was admitted for investigation due to dyspnea on minimal effort, dry cough, dysphagia, and a 12-kg weight loss over 8 months. Physical examination revealed collateral circulation in the anterior thoracic region. Laboratory tests performed at admission showed only thrombocytopenia. Chest CT revealed a bulky lobulated mass in the anterior mediastinum (Figure 1A), calcified “eggshell” lymph nodes in various mediastinal chains, and fibrotic interstitial changes consistent with chronic sarcoidosis (Figures 1B and C). PET/CT demonstrated glycolytic hypermetabolism suggesting the presence of neoplastic tissue within the mass (Figure 1D). Biopsy of the mediastinal mass with immunohistochemical analysis led to the diagnosis of B-cell lymphoma. The patient underwent a 1-year regimen of chemotherapy combined with corticosteroid treatment, which resulted in marked reduction in mediastinal mass volume and glycolytic metabolism (Figure 1E).

The diagnosis of sarcoidosis is based on clinical presentation, complementary examination findings, and biopsy. Sarcoidosis increases the risk of lymphoma development, possibly via lymphocytic proliferation associated with high mitotic activity in the active chronic form, ultimately leading to the rare sarcoidosis-lymphoma syndrome.^1^ ^-^ ^4^ The understanding of this relationship remains limited, and the condition is documented sparsely in the medical literature.
